# Epigenetic Control of the Vasopressin Promoter Explains Physiological Ability to Regulate Vasopressin Transcription in Dehydration and Salt Loading States in the Rat

**DOI:** 10.1111/jne.12371

**Published:** 2016-04-25

**Authors:** M. P. Greenwood, M. Greenwood, B. T. Gillard, S. Y. Loh, J. F. R. Paton, D. Murphy

**Affiliations:** ^1^ School of Clinical SciencesUniversity of BristolBristolUK; ^2^ Department of PhysiologyUniversity of MalayaKuala LumpurMalaysia; ^3^ School of Physiology and PharmacologyUniversity of BristolBristolUK

**Keywords:** methylation, arginine vasopressin, supraoptic nucleus, hyperosmotic, hypovolaemia

## Abstract

The synthesis of arginine vasopressin (AVP) in the supraoptic nucleus (SON) and paraventricular nucleus (PVN) of the hypothalamus is sensitive to increased plasma osmolality and a decreased blood volume, and thus is robustly increased by both dehydration (increased plasma osmolality and decreased blood volume) and salt loading (increased plasma osmolality). Both stimuli result in functional remodelling of the SON and PVN, a process referred to as functional‐related plasticity. Such plastic changes in the brain have recently been associated with altered patterns of DNA methylation at CpG (cytosine‐phosphate‐guanine) residues, a process considered to be important for the regulation of gene transcription. In this regard, the proximal *Avp* promoter contains a number of CpG sites and is recognised as one of four CpG islands for the *Avp* gene, suggesting that methylation may be regulating *Avp* transcription. In the present study, we show that, in an immortalised hypothalamic cell line 4B, the proximal *Avp* promoter is highly methylated, and treatment of these cells with the DNA methyltransferase inhibitor 5‐Aza‐2′‐deoxycytidine to demethylate DNA dramatically increases basal and stimulated *Avp* biosynthesis. We report no changes in the expression of DNA methyltransferases, *Dnmt1* and *Dnmt3a*, whereas there is decreased expression of the demethylating enzyme ten‐eleven‐translocation 2, *Tet2*, in the SON by dehydration and salt loading. We found higher methylation of the SON *Avp* promoter in dehydrated but not salt‐loaded rats. By analysis of individual CpG sites, we observed hypomethylation, hypermethylation and no change in methylation of specific CpGs in the SON *Avp* promoter of the dehydrated rat. Using reporter gene assays, we show that mutation of individual CpGs can result in altered *Avp* promoter activity. We propose that methylation of the SON *Avp* promoter is necessary to co‐ordinate the duel inputs of increased plasma osmolality and decreased blood volume on *Avp* transcription in the chronically dehydrated rat.

The neuropeptide hormone arginine vasopressin (AVP) is synthesised in magnocellular neurones of the supraoptic nucleus (SON) and paraventricular nucleus (PVN) of the hypothalamus. Increases in plasma osmolality are detected by osmosensitive neurones in circumventricular organs that provide direct excitatory inputs leading to increased *Avp* synthesis by magnocellular neurones of the SON and PVN, as well as AVP secretion from the posterior pituitary 1. An increase in plasma osmolality of only 1% is sufficient to drive increased AVP synthesis and secretion 2. Vasopressin synthesis and secretion is also sensitive to non‐osmotic cues, including changes in blood volume and pressure 3, 4, 5, 6, 7. A decrease in blood volume (hypovolaemia) is detected by the cardiac right atrium, again resulting in increased AVP synthesis and secretion 8. In this regard, changes in blood volumes greater than 8% are necessary to facilitate this response 3, 5, 9, 10. A population of smaller AVP expressing parvocellular neurones is also found in the PVN, which is important in co‐ordinating responses to stress 11.

The two osmotic stimuli of dehydration and salt loading both robustly increase *Avp* mRNA levels by approximately two‐fold in the SON and PVN, with parallel increases in the secretion of AVP 5, 9, 12. Notably, dehydration also decreases blood volume in rats, with > 20% reductions in volume by 3 days 5, 9, 13; thus, dehydration can be considered as both an osmotic and hypovolaemic stimulus. The prolonged exposure to either of these stimuli causes functional remodelling of both brain nuclei as a consequence of persistent neuronal activation, a process referred to as function‐related plasticity 14. The visible outcomes of prolonged hyperosmotic stimulation of the PVN and SON are increased volumes of magnocellular neurones and a retraction of glial processes, which is reversed upon cessation of the stimulus 14, 15. The hypertrophy of magnocellular neurones is recognised to be a result of the large increase in transcription and protein synthesis under hyperosmotic stimulation. In this regard, catalogues of differentially expressed genes have been reported in the SON and PVN in response to both dehydration and salt loading that are consistent with increased levels of transcription 16, 17, 18. These lists include the up‐regulated expression of a wide array of transcription factors that, through their interaction at the promoters of target genes, are important for this wave of increased transcriptional activity.

A previous study has suggested that brain plasticity is dependent upon epigenetic mechanisms resulting in stable modulation of gene expression 19. Indeed, a study by Guo *et al*. 20 reported that increased neuronal activity could promote vast methylation changes throughout the genome. The epigenetic modification receiving most attention is methylation of CpG (cytosine‐phosphate‐guanine) sites. The notion that the methylation of genomic DNA was stable in adult life is now discredited. The adult brain expresses high levels of DNA methyltransferases (*Dnmt*) that are involved in the active maintenance and *de novo* methylation of CpG residues in genomic DNA 21, 22. In addition, the ten‐eleven‐translocation (*Tet*) genes are also abundant in the brain and act to facilitate active demethylation at CpG residues 23. Increased methylation is commonly associated with transcriptional inhibition, although, in some cases, transcriptional activation has also been reported 24. Thus, we proposed that changes in methylation may contribute to the plastic response in the hypothalamus in response to dehydration and salt loading.

We focused our attention on the *Avp* promoter. The *Avp* gene has been the subject of a number of methylation studies in both the rat and mouse hypothalamus and other brain regions 25, 26, 27, 28. The methylation status of the mouse *Avp* gene has been comprehensively described in the PVN, where early‐life stress results in hypomethylation at CpGs sites in a putative enhancer within the intergenic region between the *Avp* gene and the gene encoding the closest related hormone oxytocin, which was shown to be consistent with increased *Avp* expression in adult animals 27. A subsequent study showed that the methylation patterns of these CpG sites were also altered by age, suggesting that alterations in methylation signatures within the *Avp* gene could be influenced by events later in life 28.

Four CpG islands have been identified for the *Avp* gene, and the so named CpG island 1 spans the proximal promoter region of the *Avp* gene 27, 29. The proximal portion of the *Avp* promoter includes a number of potential transcription factor binding sites, including activator proteins 1 and 2, cAMP responsive element (CRE) sites, and E‐box and G‐box elements 30, 31. We recently reported that CREB3L1 is a transcription factor of the *Avp* gene and, using chromatin immunoprecipitation, showed that it binds within the first few hundred bases upstream of the transcriptional start site 30. The immediate early genes *c‐Fos* and *c‐Jun*, as well as CREB, have also been implicated in induction of *Avp* transcription via interactions with this segment of DNA 31, 32, 33. Methylation was shown to inhibit transcription factor interactions with DNA either directly or through the recruitment of methyl binding proteins 34. Thus, we reasoned that the proximal promoter may be subjected to methylation changes in the hypothalamus in response to hyperosmotic stress.

We examined the methylation status of the proximal *Avp* promoter in the SON in response to the hyperosmotic stressors of dehydration and salt loading. The SON was the region of choice because it contains only magnocellular neurones, whereas the PVN is more heterogenous with respect to its neuronal phenotypes and physiological functions. We found that dehydration induces hypermethylation of the proximal *Avp* promoter, whereas the methylation status was not altered by salt loading.

## Materials and methods

### Animals

Male Sprague–Dawley rats weighing 275–300 g were used in the present study. Rats were maintained under a 14 : 10 h light/dark cycle (lights on 05.00 h) with food and water available *ad lib*. for at least 1 week prior to experimentation. Animal experiments were performed between 09.00 h and 11.00 h. To induce hyperosmotic stress, either water was removed for 3 days of dehydration or replaced by 2% (w/v) NaCl in drinking water for 7 days as a protocol for salt loading. All rats were humanely killed by striking of the cranium (stunning) and then immediately decapitated with a small animal guillotine (Harvard Apparatus, Holliston, MA, USA). Brains were rapidly removed from the cranium and frozen on dry ice (within 3 min after stunning) before being stored at −80 °C. All experiments were performed under a Home Office UK licence held under, and in strict accordance with, the provision of the UK Animals (Scientific Procedures) Act (1986); they had also been approved by the University of Bristol Animal Welfare and Ethical Review board.

### Duel extraction of DNA and RNA from the same sample

Frozen brains were sliced into 60‐μm coronal sections in a cryostat. Sections were mounted on glass slides and stained with 0.1% (w/v) toludine blue, then visualised on a light microscope until magnocellular neurones of the SON were visible. SON samples (24 punches) were collected from twelve coronal slices using a 0.35‐mm sample corer (Fine Science Tools, Foster City, CA, USA) using the optic chiasm as a reference. Punches were dispensed into 0.5‐ml tubes kept on dry ice within the cryostat. Cortex samples (four punches) were collected from two consecutive sections after SON collection using a 0.8‐mm sample corer to obtain similar quantities of tissue (Fine Science Tools). Total RNA and genomic DNA were extracted from each sample using ZR‐Duet DNA/RNA MiniPrep kit (Zymo Research, Irvine, CA, USA). The punch samples (SON and cortex) were removed from dry ice and rapidly resuspended, by vortexing, in 400 μl of DNA/RNA lysis buffer. Subsequent steps were performed in accordance with the manufacturer's instructions.

For *in vitro* studies, the culture medium was removed and the cells were lysed in 350 μl of Qiazol Reagent (Qiagen, Valencia, CA, USA). The lysate was mixed with 350 μl of absolute ethanol and added directly into the Direct‐zol™ RNA MiniPrep columns (Zymo Research; R2052) and extraction continued in accordance with the manufacturer's instructions. The concentrations of DNA and RNA were determined using a NanoDrop (Thermo Scientific, Waltham, MA, USA).

### Bisulphite conversion of DNA and TA cloning

Genomic DNA from SON and cortex punches (50 ng) and rat hypothalamic 4B cells (200 ng) was bisulphite converted using EZ DNA Methylation‐Gold kit (Zymo Research). Primers for amplification of bisulphite converted DNA were designed using methprimer 35. The converted DNA was amplified with rat *Avp* promoter primers (5′‐TTGTTGAGAGTTGTTGAAATGTTTAAT‐3′ and 5′‐TTTATATCTACAAATATTAACTAAAAAAC‐3′) using the cycling conditions: 94 °C for 2 min followed by 45 cycles of 94 °C for 30 s, 50 °C for 30 s and 72 °C for 2 min. Polymerase chain reactions (PCRs) were performed using Platinum Taq DNA Polymerase (Life Technologies, Grand Island, NY, USA). The PCR products were purified using Qiagen's PCR purification kit, ligated into pGEM‐T Easy vector (Promega, Madison, WI, USA), and transformed into DH5α competent *Escherichia coli* cells. Positive clones were selected by blue/white colony selection on liquid broth agar plates supplemented with X‐Gal and isopropyl β‐d‐1‐thiogalactopyranoside. Plasmid DNA was extracted from overnight bacterial cultures using a QIAprep Spin Miniprep kit (Qiagen) and the presence of inserts was verified by restriction digestion with *Eco*RI. Twenty independent clones were sequenced per SON sample and 10 independent clones per sample for cortex and hypothalamic 4B cells.

### Cell treatments

Rat hypothalamic 4B cells were cultured in Dulbecco's modified Eagle's medium (DMEM) (Sigma, St Louis, MO, USA; D6546) supplemented with 10% (v/v) heat‐inactivated foetal bovine serum (Gibco, Gaithersburg, MD, USA), 2 mm l‐glutamine and 100 units/ml of penicillin‐streptomycin. Cells were incubated at 37 °C in a humidified incubator with 5% (v/v) CO_2_. For chemical treatments, cells were seeded onto tissue culture plates to 60–70% confluence. After 24 h, cells were treated with 1, 2.5, 5 or 10 μm of DNA methyltransferase inhibitor 36, 5‐Aza‐2′‐deoxycytidine (5‐Aza; Sigma, A3656) for 48 h. For activation of the cAMP pathway, cells were treated with 10 μM forskolin (Sigma: F6886) or dimethyl sulphoxide (DMSO) (vehicle) for 4 h. Stock solutions of 5‐Aza (10 mm) and forskolin (10 mm) were prepared in DMSO.

### cDNA synthesis and quantitative PCR analysis

For cDNA synthesis, total RNA (50 ng for punch samples, 500 ng for hypothalamic 4B cells) was reverse transcribed using the Quantitect reverse transcription kit (Qiagen). Primers for *Avp* (5′‐TGCCTGCTACTTCCAGAACTGC‐3′ and 5′‐AGGGGAGACACTGTCTCAGCTC‐3′), heteronuclear *Avp* (*hnAvp*) (5′‐GAGGCAAGAGGGCCACATC‐3′ and 5′‐CTCTCCTAGCCCATGACCCTT‐3′), *c‐Fos* (5′‐AGCATGGGCTCCCCTGTCA‐3′ and 5′‐GAGACCAGAGTGGGCTGCA‐3′), *Creb3l1* (5′‐GCCAACAGGACCCTGCTCCA‐3′ and 5′‐AGTGCCAGTCTGTGTGGCCG‐3′), *Dnmt1* (5′‐AACCACTCAGCATTCCCGTA‐3′ and 5′‐TGCTGGTACTTCAGGTCAGG‐3′), *Dnmt3a* (5′‐AAGACCCCTGGAACTGCTAC‐3′ and 5′‐TGGCGAAGAACATCTGGAGT‐3′), *Tet1* (5′‐TGACCCACTCTTACCAGACC‐3′ and 5′‐GATGGGCCATTGCTTGATGT‐3′), *Tet2* (5′‐TCGGAGGAGAAGAGTCAGGA‐3′ and 5′TAGGGCTTGCATTTTCCATC‐3′), *Tet3* (5′‐ATGGCATGAAACCACCCAAC‐3′ and 5′‐ACTTGATCTTCCCCTCCAGC‐3′) and *Rpl19* (5′‐GCGTCTGCAGCCATGAGTA‐3′ and 5′‐TGGCATTGGCGATTTCGTTG‐3′) were synthesised by Eurofins MWG Operon (Ebersberg, Germany). The optimisation and validation of primers was performed using standard ABI protocols. The cDNA from reverse transcription reaction was diluted 1 : 4 with ddH_2_O and used as a template for subsequent PCRs, which were carried out in duplicate using SYBR green (Roche, Basel, Switzerland) on an ABI StepOnePlus Real‐Time PCR system (Applied Biosystems, Foster City, CA, USA). For relative quantification of gene expression, the 2^−ΔΔ*CT*^ method was employed 37. The internal control gene used for these analyses was the housekeeping gene Rpl19.

### Construction of *Avp* promoter mutants by overlap extension PCR

A series of mutant rat *Avp* promoter luciferase constructs were generated by overlap extension PCR. Primers 350‐F (5′‐CGGGGTACCAATGAGACCTGGGGACCCCT‐3′) and 350‐R (5′‐CCCGCTCGAGCCTGAGCGGGCTGGGCTGT‐3′) were designed to amplify 350 bp of the rat *Avp* promoter, and contained *Kpn*I and *Xho*I restriction sites, respectively. These primers were used in combination with deletion specific forward and reverse primers to amplify *Avp* promoter fragments using Phusion High‐Fidelity DNA Polymerase (New England Biolabs, Beverly, MA, USA). The PCR products from the initial PCRs were combined and used as template for a subsequent PCR using primers 350‐F and 350‐R. Mutations (C‐A) were performed for six separate CpG sites and complete 350‐bp fragments were ligated into *Kpn*I and *Xho*I sites of pGL3‐Basic plasmid (Promega). The primers used for creation of site‐directed mutations were:

CpG1 (5′‐CCCTCAAGTAGGCTCACCTCCC‐3′ and 5′‐GGGAGGTGAGCCTACTTGAGGG‐3′), CpG2 (5′‐TCACTGTGGAGGTGGCTCCCG‐3′ and 5′‐CGGGAGCCACCTCCACAGTGA‐3′),
CpG3 (5′‐CGGTGGCTCCAGTCACACGGTG‐3′ and 5′‐CACCGTGTGACTGGAGCCACCG‐3′),
CpG4 (5′‐CCCGTCACAAGGTGGCCAGTG‐3′ and 5′‐CACTGGCCACCTTGTGACGGG‐3′),
CpG6 (5′‐TTAGCAGCCAAGCTGTCGCCTCC‐3′ and 5′‐GGAGGCGACAGCTTGGCTGCTAA‐3′) and CpG7 (5′‐GCCACGCTGTAGCCTCCTAGCCA‐3′ and 5′‐TGGCTAGGAGGCTACAGCGTGGC‐3′).

### *In vitro* methylation of plasmid DNA

Methylation of *Avp* promoter constructs was performed using CpG methyltransferase (M0226L; NEB) in accordance with the manufacturer's instructions. Mock methylation reactions were performed for all plasmids with identical reaction chemistry but with the omission of CpG methyltransferase. The methylation reactions were incubated at 37 °C for 8 h followed by 65 °C for 20 min. Plasmid DNA was purified using a QIAprep Spin Miniprep kit. To confirm methylation, plasmids were cut with methylation sensitive restriction enzyme *Pml*I (CACGTG), which recognises CpG site 5 within the *Avp* promoter of the pGL3‐*Avp* promoter constructs.

### Luciferase assay

Human embryonic kidney cells HEK293T/17 (ATTC CRL‐11268) were cultured in DMEM (Sigma; D6546) supplemented with 10% (v/v) heat‐inactivated foetal bovine serum (Gibco, Gaithersburg, MD, USA), 2 mm l‐glutamine and 100 units/ml of penicillin‐streptomycin. Cells were incubated at 37 °C in a humidified incubator with 5% (v/v) CO_2_. For luciferase assays, 3 × 10^5^ cells/well were seeded in 12‐well tissue culture plates in the absence of antibiotics. The next day, plasmids (0.5 μg of pGL3‐*Avp* promoter and 0.5 μg of pcDNA3 or pcDNA3‐*Creb3l1* and 0.05 μg of pRL‐TK vector/well) were transfected with FuGENE HD transfection reagent (Promega). The culture media was replaced with fresh media at 8 h after transfection. Luciferase assays were performed using Promega's Dual‐Luciferase^®^ Reporter Assay kit. At 36 h after transfection, culture media was removed and cells were washed with phosphate‐buffered saline and lysed with 500 μl of the supplied lysis buffer. Luciferase activity was measured using a Lumat LB 9507 Luminometer (Berthold Technologies GmbH, Bad Wildbad, Germany).

### Statistical analysis

One‐way anova with Tukey's post‐hoc test were used to determine the difference between more than two samples with only a single influencing factor. Two‐way anova with a Bonferonni post‐hoc test was used to determine interactions between two independent variables on the dependent variable. In some cases, statistical differences between two experimental groups were evaluated using an independent‐sample unpaired Student's t‐test. Correlations were performed using Pearson's correlation coefficient. P < 0.05 was considered statistically significant.

## Results

### CpG sites in the proximal *Avp* promoter

**Figure 1:**
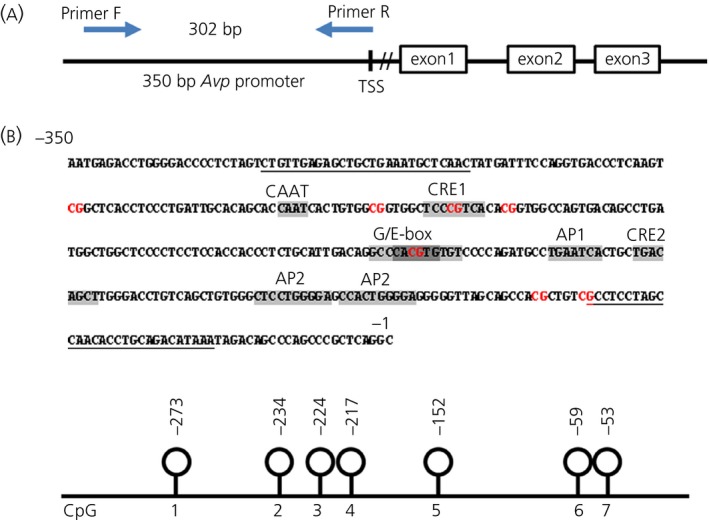
Schematic diagram of the *Avp* gene and the presence of CpG (cytosine‐phosphate‐guanine) sites in the proximal promoter region. (a) *Avp* gene contains three exons indicated by open boxes. The primers used to amplify the 302‐bp *Avp* promoter are indicated. (b) Transcription factor binding sites (highlighted) and CpG sites (red) investigated within the 350 bp of *Avp* promoter are shown. The location of forward and reverse primers for amplification of bisulphite converted DNA are underlined. Lower: location of CpG sites in the *Avp* promoter. TSS, transcription start site; CAAT box; CRE, cAMP response element; AP, activator protein.

The *Avp* gene is comprised of three exons and two introns (Fig. 1 a). Computational studies mapping the distribution of CpG residues throughout the *Avp* gene have described the location of a CpG island in the proximal Avp promoter region 27. We focused our attention on seven CpG residues in this CpG island (Fig. 1 b). This region of the Avp promoter contains a number of transcription factor binding motifs.

### Demethylation of the *Avp* promoter dramatically increases *Avp* transcription

**Figure 2:**
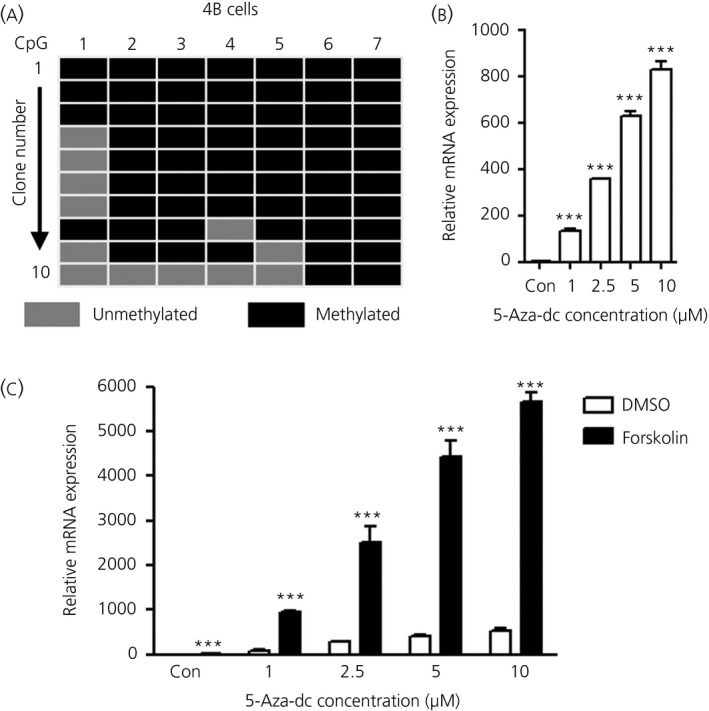
Demethylation of the *Avp* promoter dramatically increases *Avp* transcription in hypothalamic 4B cells. (a) Tile diagram showing the methylation status of CpG (cytosine‐phosphate‐guanine) sites for individual clones of the *Avp* promoter from the hypothalamic 4B cells. (b) Treatment of hypothalamic 4B cells with DNA methyltransferase inhibitor 5‐Aza‐dc (1–10 μm) increases *Avp* synthesis. (c) Forskolin (10 μm) induced *Avp* synthesis was further enhanced by 5‐Aza treatment. Error bars indicate the mean ± SEM (n = 4 per group). ***P < 0.001 (b, one‐way anova with Tukey's post‐hoc test; c, two‐way anova with a Bonferonni post‐hoc test). DMSO, dimethyl sulphoxide.

We first looked at *Avp* promoter methylation in an immortalised rat hypothalamic cell line 4B. Hypothalamic 4B cells are derived from embryonic day 19 rat hypothalamus and have a neuronal phenotype expressing corticotrophin‐releasing hormone, *Avp* and glucocorticoid receptors similar to parvocellular neurones of the hypothalamus 38. We found high methylation of the *Avp* promoter region in hypothalamic 4B cells (Fig. 2 a). Treatment of hypothalamic 4B cells with the DNA methyltransferase inhibitor 5‐Aza dramatically increased *Avp* expression (Fig. 2 b). We treated cells with 5‐Aza in the presence or absence of forskolin (Fig. 2 c). Forskolin alone increased *Avp* synthesis and further enhanced responses to 5‐Aza treatment, suggesting that methylation regulates cAMP induced *Avp* synthesis.

### Decreased expression of Tet2 in the SON of dehydrated and salt‐loaded rats

**Figure 3:**
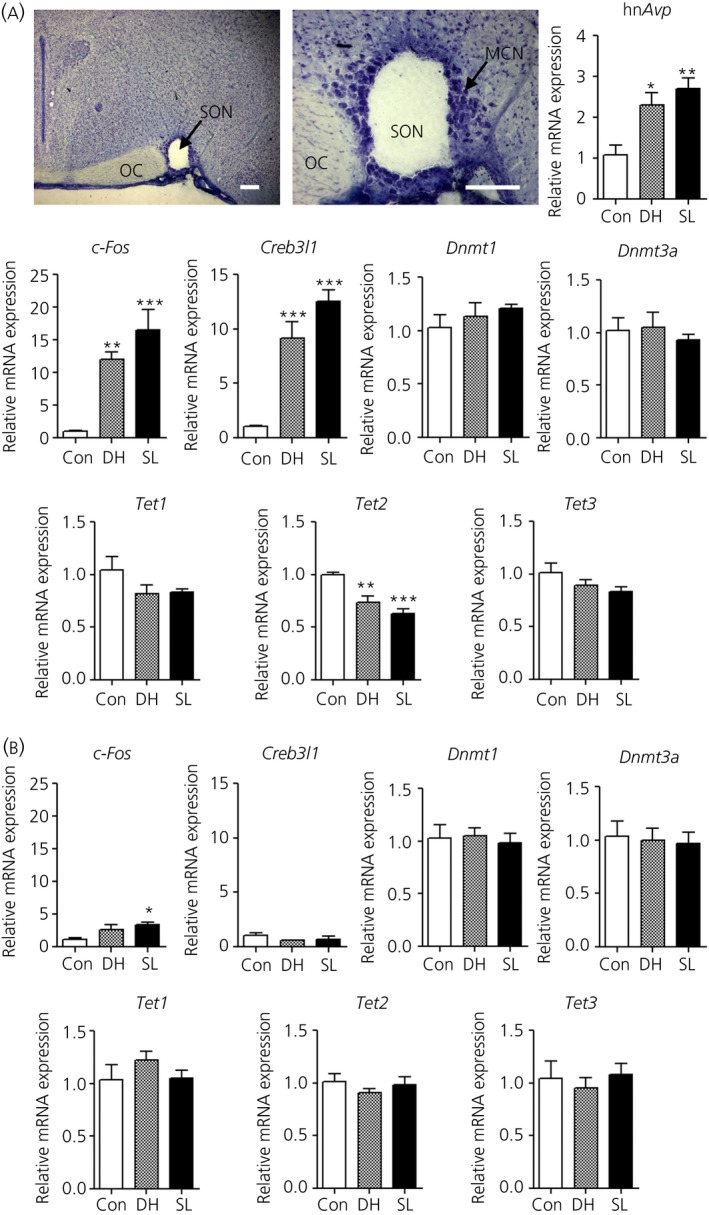
mRNA expression of genes involved in hyperosmotic stress and methylation in the supraoptic nucleus (SON) and cortex of dehydrated and salt‐loaded rats. (a) Brain section stained with 1% (w/v) toludine blue/70% (v/v) ethanol shows the punched area of the SON. The high magnification image shows that the punch samples were confined within the area of magnocellular neurones of the SON. cDNA synthesis and subsequent quantitative polymerase chain reaction analysis was performed using RNA extracted from these punch samples. mRNA expression of hn*Avp*,* c‐Fos*,* Creb3l1*,* Dnmt1*,* Dnmt3a*,* Tet1*,* Tet2* and *Tet3* was examined in SON (a) and cortex (b). Error bars indicate the mean ± SEM (n = 4–5 per group). *P < 0.05, **P < 0.01, ***P < 0.001 (one‐way anova with Tukey's post‐hoc test). OC, optic chiasm; MCN, magnocellular neurone; DH, dehydration; SL, salt loading; hn*Avp*, heteronuclear RNA of *Avp*. Scale bar = 200 μm.

We used a micro punch to isolate SON samples aiming to minimise contamination from surrounding brain regions (Fig. 3). Our data replicate previous findings showing that *hnAvp* expression is robustly induced in the SON by the two osmotic stimuli of dehydration and salt loading (Fig. 3 a) 5. As expected, using immediate early gene *c‐Fos* (Fig. 3 a), a marker of neuronal activity, we confirmed comparable increases in neuronal activity in both dehydrated and salt‐loaded rat SON. We have recently identified transcription factor CREB3L1 as a putative transcriptional regulator of the *Avp* gene and confirm here that *Creb3l1* is induced by dehydration and salt loading (Fig. 3 a) 30. To determine whether changes in methylation may underlie the increased transcription of *Avp*, we looked at the mRNA expression of *Dnmt* and *Tet* genes in the SON (Fig. 3 a). We found no changes in expression of *Dnmt1*,* Dnmt3a*,* Tet1* or *Tet3* in the SON of dehydrated or salt‐loaded rats compared to controls. By contrast, *Tet2* expression was significantly decreased in SON by both stimuli. We chose to examine gene expression in an unrelated brain region, the cortex (Fig. 3 b). In this brain region, *Avp* expression was undetectable, and there were no differences in the expression of any of the genes examined compared to controls, with the exception of increased *c‐Fos* in salt‐loaded animals.

### Change in methylation status of the *Avp* promoter in the dehydrated rat

**Figure 4:**
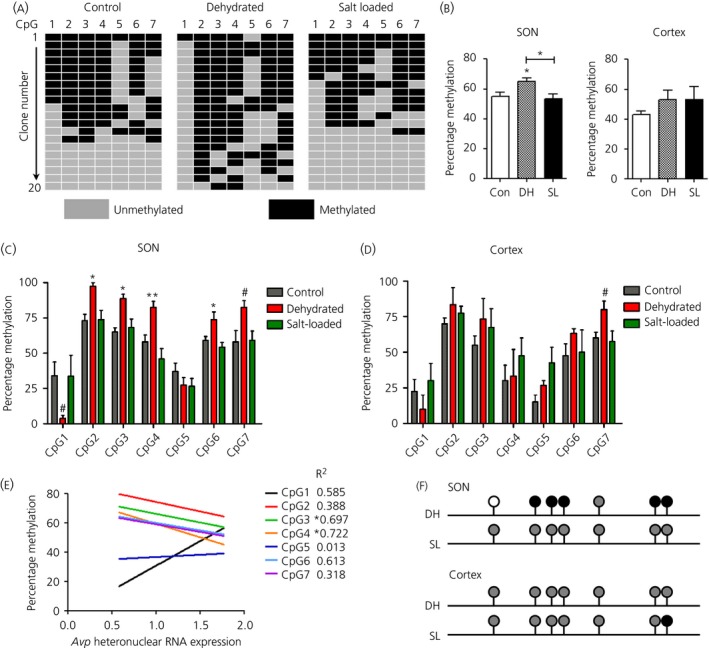
Methylation status of the *Avp* promoter in response to dehydration and salt loading in the supraoptic nucleus (SON). Genomic DNA was extracted from the SON and cortex of control, 3 days dehydrated and 7 days salt‐loaded rats (n = 4–5). The DNA was bisulphite converted, *Avp* promoter region amplified and cloned into TA vector for sequencing (n = 20 for each sample). (a) Tile diagrams showing the methylation status of seven CpG (cytosine‐phosphate‐guanine) sites for individual clones of the *Avp* promoter extracted from the SON. (b) Percentage of global methylation on the *Avp* promoter in the SON and cortex of dehydrated and salt‐loaded rats is shown. (c, d) Change in methylation status of CpGs in (c) SON and (d) cortex *Avp* promoters in response to dehydration and salt loading. (e) Correlation analysis of methylation level with the expression of hn*Avp* in control SON samples. (f) Methylation level of CpG 1–7 on the *Avp* promoter of dehydrated and salt‐loaded rats compared to control. Black, hypermethylation; white, hypomethylation; grey, no change in methylation level compared to control. Error bars indicate the mean ± SEM (n = 4–5 per group). *P < 0.05, **P < 0.01 (one‐way anova with Tukey's post‐hoc test); ^#^P < 0.05 (unpaired t‐test); DH, dehydration; SL, salt loading.

We examined the methylation profile of the *Avp* promoter within the SON by sequence analysis of bisulphite converted DNA (Fig. 4). Using primers spanning the proximal *Avp* promoter (−325 to −24), we investigated the methylation status of this cluster of seven CpG sites. Analyses of the methylation pattern of CpGs in single clones from individual control, dehydrated and salt‐loaded animals are depicted in Fig. 4(a). These data showed that all CpGs in this region have some degree of methylation under all experimental conditions. Analysis of the overall methylation of the *Avp* promoter for the SON revealed increased methylation in dehydrated compared to control and salt‐loaded animals (Fig. 4 b). In comparison, methylation was not significantly affected by dehydration or salt loading in the cortex (Fig. 4 b).

We next compared the methyation profiles of the three experimental groups. Of the seven CpGs analysed, five showed increased methylation (CpGs 2, 3, 4, 6 and 7), one showed decreased methylation (CpG1) and one showed no change of methylation (CpG5) in the SON of dehydrated compared to control animals (Fig. 4 c). By contrast, methylation of the *Avp* promoter was not altered in the SON by salt loading. To determine whether these changes were specific to the SON, we used the same method to look at *Avp* promoter methylation in the cortex of these animals (Fig. 4 d). Of note, the pattern of methylation displayed across these CpGs was remarkably similar in the SON and cortex of controls, suggesting that this pattern was not unique to the SON. By contrast to the SON, dehydration only significantly influenced methylation of CpG7 in the *Avp* promoter in the cortex. However, salt loading also had no affect on methylation in the cortex.

We performed correlation analyses, comparing the methylation status of individual CpG residues with the expression of hn*Avp* in the same control sample (Fig. 4 e). Of the six CpGs that showed significant changes in methylation status in dehydration, only two CpGs (CpG3 and 4) had methylation patterns that were strongly correlated with the hn*Avp* expression in control animals. The strong negative correlation between *Avp* promoter methylation level and *Avp* hnRNA abundance is consistent with a role for methylation in transcriptional inhibition. In dehydrated and salt‐loaded animals, we observed no correlation between methylation of individual CpGs and hn*Avp* expression. A summary of the overall methylation changes is depicted in Fig. 4(f).

### *In vitro* methylation of CpG sites in the *Avp* promoter alters promoter activity

**Figure 5:**
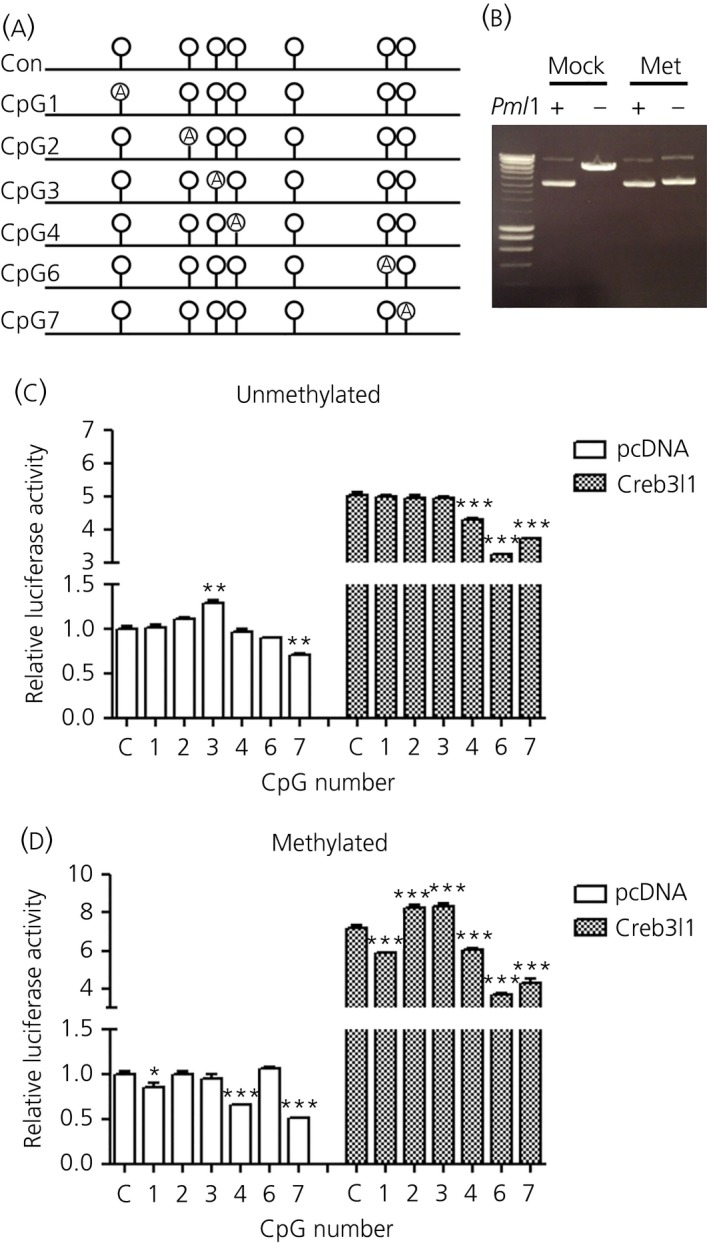
Methylation of CpG (cytosine‐phosphate‐guanine) sites on the *Avp* promoter *in vitro*. The substitution (C‐A) at CpG sites in the *Avp* promoter by overlap extension polymerase chain reaction was used to prevent methylation at specific CpG sites. The mutation sites are shown in (a). The mutated plasmids were subsequently methylated by methyltransferase enzyme. (b) Successful methylation was determined using methylation sensitive restriction enzyme *Pml*I. (c, d) Luciferase assays were performed by co‐transfection of plasmid expressing *Creb3l1* and 350 bp *Avp* promoter contructs with (c) unmethylated and (d) methylated plasmid. Error bars indicate the mean ± SEM (n = 4 per group). *P < 0.05; **P < 0.01; ***P < 0.001 (one‐way anova with Tukey's post‐hoc test). 5‐Aza‐dc, 5‐Aza‐2′‐deoxycytidine.

We then used *Avp* promoter‐luciferase reporter assays to further explore the effect of methylation on *Avp* transcription (Fig. 5). Using the technique of overlap extension PCR, we replaced the nucleotide cytosine with adenine (C‐A) for CpG sites 1, 2, 3, 4, 6, 7 aiming to prevent *in vitro* methylation of these residues by CpG methyltransferase (Fig. 5 a). We did not examine CpG5 because the methylation status of this site was not altered in the SON by dehydration or salt loading. The methylation status of our promoter constructs was confirmed by a failure to digest the plasmid DNA with *Pml*I (Fig. 5 b). We performed luciferase assays to test whether manipulation of these single nucleotides altered *Avp* promoter activity (Fig. 5 c). The C‐A substitution at CpG3, which resides in the CRE site, increased *Avp* promoter activity, whereas, at the more proximal CpG7 site, *Avp* promoter activity decreased. Next, we transfected HEK293T cells with transcription factor *Creb3l1* overexpression construct to test whether induced *Avp* transcription was altered by any of these manipulations. For CpG4, 6 and 7, C‐A substitution reduced *Creb3l1* mediated transcriptional activation of the *Avp* promoter, suggesting that those nucleotides are important for transcriptional activation by *Creb3l1*. We then investigated the effect of *in vitro* methylation by CpG metyltransferase on *Avp* promoter activity of these constructs (Fig. 5 d). After methylation, CpG1, 4 and 7 constructs displayed lower promoter activity, suggesting that the methylation status of these sites was intrinsically important for *Avp* promoter activity. In response to *Creb3l1*, methylation of CpG1, 4, 6 and 7 constructs decreased, whereas CpG2 and 3 increased, promoter activity.

## Discussion

It has been suggested that epigenetic mechanisms underlie brain plasticity 19, 20, 39, a process in which stable modulation of gene expression occurs. In this regard, the SON represents a perfect model because it undergoes huge plastic changes in response to dehydration and salt loading to cope with the increased demand for *Avp* synthesis 14. Thus, we proposed that the *Avp* gene maybe a target for epigenetic regulation in osmotic stress. The clustering of CpGs upstream of the transcriptional start site of *Avp*, a region that contains transcription factor binding motifs, implied that epigenetic modification of this segment of DNA maybe important for modulation of *Avp* transcription. We show that demethylation of the *Avp* promoter by 5‐Aza treatment of hypothalamic 4B cells greatly increases basal and stimulated *Avp* synthesis, suggesting that methylation acts to inhibit *Avp* synthesis. We also show DNA demethylation and *de novo* methylation of CpG residues in the proximal *Avp* promoter in the dehydrated, but not the salt‐loaded, rat SON.

We used hypothalamic 4B cells to investigate *Avp* promoter methylation *in vitro*. These cells have widely been used to study both corticotrophin‐releasing hormone and *Avp* transcription 38, 40, 41. Hypothalamic 4B cells were found to express *Avp* and treatment with the DNA methyltransferase inhibitor 5‐Aza dramatically increased *Avp* synthesis by forskolin treatment. Demethylation of gene promoter regions has been shown to increase the accessibility of DNA for activation by transcription factors 34. Notably, expression of house keeping gene *Rpl19* was not affected by treatment with 5‐Aza. Therefore, this cell line represents a useful model for the future investigation of transcriptional regulation of the *Avp* gene by methylation.

Increased neuronal activity has been linked with global methylation changes in the brain. In mouse dentate gyrus neurones, activation was shown to be associated with active demethylation or *de novo* methylation of specific genes related to neuronal plasticity 20. It well known that *c‐Fos*, a marker of neuronal activity, is dramatically increased in abundance in magnocellular neurones of the SON by hyperosmotic stress 42, 43, as indicated in the present study by increased mRNA expression. With this in mind, we investigated mRNA expression of genes regulating DNA methylation processes in the SON. The expression of DNA methyltransferases, *Dnmt1* and *Dnmt3a*, has been described in the adult brain 21, and we observed the expression of these genes in the SON, although levels were not altered by dehydration or salt loading. Nevertheless, one member of *Tet* family, *Tet2*, known to be involved in active and passive demethylation of DNA 23, was altered by both stimuli in the SON. Interestingly, the knockdown of *Tet2* expression *in vitro* has been associated with hypermethylation of DNA 44, 45. Therefore, we reasoned that decreased *Tet2* may result in hypermethylation of CpG sites in the SON *Avp* promoter.

The only report of methylation status regarding the proximal *Avp* promoter region was in the mouse PVN, where sparse methylation was reported 27. Indeed, previous studies on methylation of the *Avp* gene have examined regions outside of the proximal promoter, focusing on more distal extremities of the promoter or on an enhancer within the intergenic region linking the *Avp* and oxytocin genes 25, 26, 27. No study has sought to clarify the steady‐state methylation status of the *Avp* gene in magnocellular neurones of the PVN or in the SON. We have addressed this issue in the present study. To our surprise, methylation increased at CpG2, 3, 4, 6 and 7 only in dehydrated animals, highlighting that methylation of these CpGs is unstable. The fact that decreased Tet2 expression was observed in SONs of both dehydrated and salt‐loaded animals suggests that Tet2 may not be responsible for increased *Avp* promoter methylation in dehydration.

Notably, methylation levels were higher at CpG3, which resides in the middle of the CRE, and at CpG2 and CpG4, which flank this motif. Methylation at CRE sites has been shown to inhibit CREB mediated transcription 46, 47. Furthermore, promoter deletion studies in cell line JEG3 showed that deletion of both CRE sites significantly reduced forskolin stimulation of the *Avp* promoter 33. We have previously reported *Creb3l1* as a transcription factor of the *Avp* gene in the rat hypothalamus 30. A G‐box element (GCCCACGTGTGT) that flanks the previously identified E‐box enhancer element (CACGTG) was identified as a putative binding site for CREB3L1. When we deleted the core nucleotides (ACGT) from this motif, which corresponds to CpG5, *Avp* promoter activity dropped significantly, confirming the importance of this site in *Avp* promoter activity. It is interesting that the methylation status of CpG5 was the only CpG not affected by dehydration in the SON, perhaps suggesting the stringent regulation of this site for appropriate *Avp* transcription. The increased methylation of individual CpGs may alter promoter activity by interfering with the DNA binding sites of proteins that affect *Avp* transcription. Increased promoter methylation is commonly associated with transcriptional silencing, although there is evidence that methylation can also stimulate gene expression 24, 34. What we do know is that demethylation of the *Avp* promoter *in vitro* increases *Avp* expression, suggesting that methylation silences the *Avp* gene. In agreement, CpG methylation at sites 3 and 4 inversely correlated with Avp expression in control SON samples, suggesting that DNA methylation acts to confer *Avp* gene silencing *in vivo*. It is important to note that, in dehydration, *Avp* promoter methylation and *Avp* expression both increased in the SON. Therefore, we cannot rule out the possibility of methylation having a positive influence on *Avp* transcription *in vivo*.

In salt‐loaded animals, there were no changes in *Avp* promoter methylation in the SON. One explanation could be the physiological differences between these two osmotic stimuli. We recently compared the physiological and transcriptome responses to dehydration and salt loading in the rat SON 16. In the present study, we identified several fundamental differences between dehydration and salt loading in terms of natriuresis, drinking behaviour and circulating hormone levels. Despite these differences, we found that the transcriptional response by the SON was very stable, leading to the hypothesis that the SON may not be able to discriminate between these two osmotic cues. Indeed, both dehydration and salt loading result in significant increases in plasma osmolality compared to controls, although we found no difference when comparing dehydration with salt loading 16.

In the absence of drinking fluid, both extracellular and intracellular fluid volumes decrease, leading to increased plasma osmolality and, unlike salt loading, hypovolaemia 48, 49. Acute hypovolaemia, which can be caused by haemorrhaging or, pharmacologically, by polyethylene glycol injection into the rat, increases AVP secretion from the posterior pituitary, and this is accompanied by increased synthesis of *Avp* in the PVN and SON 3, 5, 9 in the absence of osmotic stimulation 3. A decrease in plasma volume of approximately 15–20% has been shown to increase *Avp* synthesis by approximately two‐fold in the SON and PVN in acute experiments. This raises the question of why the two stimuli (i.e. dehydration and salt loading) similarly increase *Avp* mRNA levels by approximately two‐fold. Indeed, a greater increase in *Avp* synthesis would be predicted in dehydrated compared to salt‐loaded animals in response to both increased plasma osmolality, as well as hypovolaemia provoked by this stimulus. Therefore, the formation of new epigenetic marks on the *Avp* promoter in dehydration may act to control synthesis of *Avp*, which is being driven by a combination of increased plasma osmolality and hypovolaemia. In support of this concept, a study of sustained hypovolaemia produced by injection of the diuretic furosemide showed that plasma AVP was increased by 8 h but decreased by 32 h, despite further decreases in blood volume 10. It was proposed that this decrease in plasma AVP may be a result of an adaptive resetting of volume control mechanisms. Unfortunately, *Avp* mRNA expression was not reported. However, Hayashi *et al*. 9 showed that acute hypovolaemia can still induce *Avp* synthesis in the PVN and SON of rats dehydrated for 3 days. This ability to increase *Avp* expression may underlie the differing mechanisms governing chronic versus acute hypovolaemia 10.

The differences in plasma AVP, oxytocin and angiotensin II levels between dehydration and salt loading 16 may also influence DNA methylation. It has been suggested that the methylation status of the *Avp* gene is maintained by the hormonal environment 25. Castration of rats was shown to increase methylation of two CpGs in the bed nucleus of the stria terminalis *Avp* promoter. This methylation was successfully reversed by the administration of testosterone. We have previously described differences in circulating angiotensin II in dehydrated and salt‐loaded animals 16 and there is evidence that angiotensin II influences methylation 50, although any effects on methylation patterns in the brain are not known. The circumventricular organs, such as the subfornical organ (SFO) and organum vasculosum lamina terminalis (OVLT), lack a functional blood–brain barrier and thus are sensitive to changes in circulating hormones 49. The SFO and OVLT expresses angiotensin II type 1 receptor 51 and the expression of this receptor is increased in the SFO by dehydration 52. The SFO directly projects to AVP expressing magnocellular neurones in the SON and electrophysical studies have shown that stimulation of the SFO affects AVP neurones 53. Therefore, it is possible that the increased circulating levels of angiotensin II in dehydration, via activation of angiotensin II type 1 receptor in the SFO, may influence *Avp* methylation events in the SON.

One of the limitations of investigating DNA methylation patterns in complex tissues such as the brain is the number of different cellular phenotypes in punch samples. In the SON, there are primarily two neuronal phenotypes (either *Avp* or oxytocin), although this nucleus also contains glial and vascular cellular phenotypes. Consequently, DNA will be extracted from all these cell types and thus contributes to the *Avp* promoter methylation profiles in the present study. Although it is not possible to state in which cell types these promoter changes occur, we observed a strong correlation at specific methylation landmarks with heteronuclear *Avp* expression in control animals, which implied that the methylation profiles may be important in maintaining steady‐state *Avp* expression in the SON. We also showed, by mutation of individual CpG sites, that these residues are important for regulating *Avp* promoter activity in cell lines.

Children and adolescents fail to completely replete their hydrational needs by drinking water or other fluids. This has led some to speculate that, over the long‐term, such deficiencies may result in a low level of dehydration and perhaps even hypovolaemia, with potential consequences later in life 54. Indeed, in the elderly, disturbances in serum sodium and blood volume are major causes of hospital admissions 55. Our data suggest that epigenetic regulation of the proximal *Avp* promoter may be an important mechanism in controlling the *Avp* synthesis in response to dehydration. We speculate that this extra level of regulation may be necessary to co‐ordinate the dual inputs received in this nucleus derived from increased plasma osmolality and hypovolaemia provoked by decreased blood volume in chronic dehydration. The stability or indeed the longevity of these newly established epigenetic marks has yet to be determined. The striking differences in methylation patterns that exist in the *Avp* promoter of dehydrated and salt‐loaded rat are likely to be important for furthering our understanding of *Avp* transcriptional control.

## References

[1] Bourque CW . Osmoregulation of vasopressin neurons: a synergy of intrinsic and synaptic processes. Prog Brain Res 1998; 119: 59–76. 10074781 10.1016/s0079-6123(08)61562-9

[2] Arima H , Kondo K , Kakiya S , Nagasaki H , Yokoi H , Yambe Y , Murase T , Iwasaki Y , Oiso Y . Rapid and sensitive vasopressin heteronuclear RNA responses to changes in plasma osmolality. J Neuroendocrinol 1999; 11: 337–341. 10320560 10.1046/j.1365-2826.1999.00308.x

[3] Dunn FL , Brennan TJ , Nelson AE , Robertson GL . The role of blood osmolality and volume in regulating vasopressin secretion in the rat. J Clin Investig 1973; 52: 3212–3219. 4750450 10.1172/JCI107521 PMC302597

[4] Kakiya S , Arima H , Yokoi H , Murase T , Yambe Y , Oiso Y . Effects of acute hypotensive stimuli on arginine vasopressin gene transcription in the rat hypothalamus. Am J Physiol Endocrinol Metab 2000; 279: E886–E892. 11001772 10.1152/ajpendo.2000.279.4.E886

[5] Kondo N , Arima H , Banno R , Kuwahara S , Sato I , Oiso Y . Osmoregulation of vasopressin release and gene transcription under acute and chronic hypovolemia in rats. Am J Physiol Endocrinol Metab 2004; 286: E337–E346. 14613925 10.1152/ajpendo.00328.2003

[6] Laycock JF , Penn W , Shirley DG , Walter SJ . The role of vasopressin in blood pressure regulation immediately following acute haemorrhage in the rat. J Physiol 1979; 296: 267–275. 529092 10.1113/jphysiol.1979.sp013004 PMC1279077

[7] Stricker EM , Verbalis JG . Interaction of osmotic and volume stimuli in regulation of neurohypophyseal secretion in rats. Am J Physiol 1986; 250: R267–R275. 3946641 10.1152/ajpregu.1986.250.2.R267

[8] Share L . Role of vasopressin in cardiovascular regulation. Physiol Rev 1988; 68: 1248–1284. 3054948 10.1152/physrev.1988.68.4.1248

[9] Hayashi M , Arima H , Goto M , Banno R , Watanabe M , Sato I , Nagasaki H , Oiso Y . Vasopressin gene transcription increases in response to decreases in plasma volume, but not to increases in plasma osmolality, in chronically dehydrated rats. Am J Physiol Endocrinol Metab 2006; 290: E213–E217. 16144818 10.1152/ajpendo.00158.2005

[10] Iwasaki Y , Gaskill MB , Robertson GL . Adaptive resetting of the volume control of vasopressin secretion during sustained hypovolemia. Am J Physiol 1995; 268: R349–R357. 7864228 10.1152/ajpregu.1995.268.2.R349

[11] Engelmann M , Landgraf R , Wotjak CT . The hypothalamic‐neurohypophysial system regulates the hypothalamic‐pituitary‐adrenal axis under stress: an old concept revisited. Front Neuroendocrinol 2004; 25: 132–149. 15589266 10.1016/j.yfrne.2004.09.001

[12] Yue C , Mutsuga N , Sugimura Y , Verbalis J , Gainer H . Differential kinetics of oxytocin and vasopressin heteronuclear RNA expression in the rat supraoptic nucleus in response to chronic salt loading in vivo. J Neuroendocrinol 2008; 20: 227–232. 18088359 10.1111/j.1365-2826.2007.01640.x

[13] Gardiner SM , Bennett T . Interactions between neural mechanisms, the renin‐angiotensin system and vasopressin in the maintenance of blood pressure during water deprivation: studies in Long Evans and Brattleboro rats. Clin Sci (Lond) 1985; 68: 647–657. 2485266 10.1042/cs0680647

[14] Hatton GI . Function‐related plasticity in hypothalamus. Annu Rev Neurosci 1997; 20: 375–397. 9056719 10.1146/annurev.neuro.20.1.375

[15] Hatton GI , Walters JK . Induced multiple nucleoli, nucleolar margination, and cell size changes in supraoptic neurons during dehydration and rehydration in the rat. Brain Res 1973; 59: 137–154. 4747747 10.1016/0006-8993(73)90256-4

[16] Greenwood MP , Mecawi AS , Hoe SZ , Mustafa MR , Johnson KR , Al‐Mahmoud GA , Elias LL , Paton JF , Antunes‐Rodrigues J , Gainer H , Murphy D , Hindmarch CC . A comparison of physiological and transcriptome responses to water deprivation and salt loading in the rat supraoptic nucleus. Am J Physiol Regul Integr Comp Physiol 2015; 308: R559–R568. 25632023 10.1152/ajpregu.00444.2014 PMC4386000

[17] Hindmarch C , Yao S , Beighton G , Paton J , Murphy D . A comprehensive description of the transcriptome of the hypothalamoneurohypophyseal system in euhydrated and dehydrated rats. Proc Natl Acad Sci USA 2006; 103: 1609–1614. 16432224 10.1073/pnas.0507450103 PMC1360533

[18] Johnson KR , Hindmarch CC , Salinas YD , Shi Y , Greenwood M , Hoe SZ , Murphy D , Gainer H . A RNA‐seq analysis of the rat supraoptic nucleus transcriptome: effects of salt loading on gene expression. PLoS One 2015; 10: e0124523. 25897513 10.1371/journal.pone.0124523 PMC4405539

[19] Flavell SW , Greenberg ME . Signaling mechanisms linking neuronal activity to gene expression and plasticity of the nervous system. Annu Rev Neurosci 2008; 31: 563–590. 18558867 10.1146/annurev.neuro.31.060407.125631 PMC2728073

[20] Guo JU , Ma DK , Mo H , Ball MP , Jang MH , Bonaguidi MA , Balazer JA , Eaves HL , Xie B , Ford E , Zhang K , Ming GL , Gao Y , Song H . Neuronal activity modifies the DNA methylation landscape in the adult brain. Nat Neurosci 2011; 14: 1345–1351. 21874013 10.1038/nn.2900 PMC3183401

[21] Auger CJ , Auger AP . Permanent and plastic epigenesis in neuroendocrine systems. Front Neuroendocrinol 2013; 34: 190–197. 23707698 10.1016/j.yfrne.2013.05.003

[22] Brown SE , Weaver IC , Meaney MJ , Szyf M . Regional‐specific global cytosine methylation and DNA methyltransferase expression in the adult rat hippocampus. Neurosci Lett 2008; 440: 49–53. 18539393 10.1016/j.neulet.2008.05.028

[23] Guo JU , Su Y , Zhong C , Ming GL , Song H . Emerging roles of TET proteins and 5‐hydroxymethylcytosines in active DNA demethylation and beyond. Cell Cycle 2010; 10: 2662–2668. 21811096 10.4161/cc.10.16.17093 PMC3219536

[24] Metivier R , Gallais R , Tiffoche C , Le Peron C , Jurkowska RZ , Carmouche RP , Ibberson D , Barath P , Demay F , Reid G , Benes V , Jeltsch A , Gannon F , Salbert G . Cyclical DNA methylation of a transcriptionally active promoter. Nature 2008; 452: 45–50. 18322525 10.1038/nature06544

[25] Auger CJ , Coss D , Auger AP , Forbes‐Lorman RM . Epigenetic control of vasopressin expression is maintained by steroid hormones in the adult male rat brain. Proc Natl Acad Sci USA 2011; 108: 4242–4247. 21368111 10.1073/pnas.1100314108 PMC3053981

[26] Bowen MT , Dass SA , Booth J , Suraev A , Vyas A , McGregor IS . Active coping toward predatory stress is associated with lower corticosterone and progesterone plasma levels and decreased methylation in the medial amygdala vasopressin system. Horm Behav 2014; 66: 561–566. 25127982 10.1016/j.yhbeh.2014.08.004

[27] Murgatroyd C , Patchev AV , Wu Y , Micale V , Bockmuhl Y , Fischer D , Holsboer F , Wotjak CT , Almeida OF , Spengler D . Dynamic DNA methylation programs persistent adverse effects of early‐life stress. Nat Neurosci 2009; 12: 1559–1566. 19898468 10.1038/nn.2436

[28] Murgatroyd C , Wu Y , Bockmuhl Y , Spengler D . The Janus face of DNA methylation in aging. Aging 2012; 2: 107–110. 20354272 10.18632/aging.100124 PMC2850147

[29] Weber M , Hellmann I , Stadler MB , Ramos L , Paabo S , Rebhan M , Schubeler D . Distribution, silencing potential and evolutionary impact of promoter DNA methylation in the human genome. Nat Genet 2007; 39: 457–466. 17334365 10.1038/ng1990

[30] Greenwood M , Bordieri L , Greenwood MP , Rosso Melo M , Colombari DS , Colombari E , Paton JF , Murphy D . Transcription factor CREB3L1 regulates vasopressin gene expression in the rat hypothalamus. J Neurosci 2014; 34: 3810–3820. 24623760 10.1523/JNEUROSCI.4343-13.2014 PMC3951688

[31] Yoshida M . Gene regulation system of vasopressin and corticotropin‐releasing hormone. Gene Regul Syst Bio 2008; 2: 71–88. 10.4137/grsb.s424 PMC2733102 19787076

[32] Carter DA , Murphy D . Regulation of c‐fos and c‐jun expression in the rat supraoptic nucleus. Cell Mol Neurobiol 1990; 10: 435–445. 2123746 10.1007/BF00711185 PMC11567296

[33] Iwasaki Y , Oiso Y , Saito H , Majzoub JA . Positive and negative regulation of the rat vasopressin gene promoter. Endocrinology 1997; 138: 5266–5274. 9389510 10.1210/endo.138.12.5639

[34] Klose RJ , Bird AP . Genomic DNA methylation: the mark and its mediators. Trends Biochem Sci 2006; 31: 89–97. 16403636 10.1016/j.tibs.2005.12.008

[35] Li LC , Dahiya R . MethPrimer: designing primers for methylation PCRs. Bioinformatics 2002; 18: 1427–1431. 12424112 10.1093/bioinformatics/18.11.1427

[36] Goffin J , Eisenhauer E . DNA methyltransferase inhibitors‐state of the art. Ann Oncol 2002; 13: 1699–1716. 12419742 10.1093/annonc/mdf314

[37] Livak KJ , Schmittgen TD . Analysis of relative gene expression data using real‐time quantitative PCR and the 2(‐Delta Delta C(T)) Method. Methods 2001; 25: 402–408. 11846609 10.1006/meth.2001.1262

[38] Kasckow J , Mulchahey JJ , Aguilera G , Pisarska M , Nikodemova M , Chen HC , Herman JP , Murphy EK , Liu Y , Rizvi TA , Dautzenberg FM , Sheriff S . Corticotropin‐releasing hormone (CRH) expression and protein kinase A mediated CRH receptor signalling in an immortalized hypothalamic cell line. J Neuroendocrinol 2003; 15: 521–529. 12694378 10.1046/j.1365-2826.2003.01026.x

[39] Azzi A , Dallmann R , Casserly A , Rehrauer H , Patrignani A , Maier B , Kramer A , Brown SA . Circadian behavior is light‐reprogrammed by plastic DNA methylation. Nat Neurosci 2014; 17: 377–382. 24531307 10.1038/nn.3651

[40] Kageyama K , Hanada K , Iwasaki Y , Sakihara S , Nigawara T , Kasckow J , Suda T . Pituitary adenylate cyclase‐activating polypeptide stimulates corticotropin‐releasing factor, vasopressin and interleukin‐6 gene transcription in hypothalamic 4B cells. J Endocrinol 2007; 195: 199–211. 17951532 10.1677/JOE-07-0125

[41] Kageyama K , Kumata Y , Akimoto K , Takayasu S , Tamasawa N , Suda T . Ghrelin stimulates corticotropin‐releasing factor and vasopressin gene expression in rat hypothalamic 4B cells. Stress 2011; 14: 520–529. 21438782 10.3109/10253890.2011.558605

[42] Chan RK , Brown ER , Ericsson A , Kovacs KJ , Sawchenko PE . A comparison of two immediate‐early genes, c‐fos and NGFI‐B, as markers for functional activation in stress‐related neuroendocrine circuitry. J Neurosci 1993; 13: 5126–5138. 8254363 10.1523/JNEUROSCI.13-12-05126.1993 PMC6576398

[43] Ji LL , Fleming T , Penny ML , Toney GM , Cunningham JT . Effects of water deprivation and rehydration on c‐Fos and FosB staining in the rat supraoptic nucleus and lamina terminalis region. Am J Physiol Regul Integr Comp Physiol 2005; 288: R311–R321. 15458969 10.1152/ajpregu.00399.2004

[44] Grosser C , Wagner N , Grothaus K , Horsthemke B . Altering TET dioxygenase levels within physiological range affects DNA methylation dynamics of HEK293 cells. Epigenetics 2015; 10: 819–833. 26186463 10.1080/15592294.2015.1073879 PMC4622922

[45] Rasmussen KD , Jia G , Johansen JV , Pedersen MT , Rapin N , Bagger FO , Porse BT , Bernard OA , Christensen J , Helin K . Loss of TET2 in hematopoietic cells leads to DNA hypermethylation of active enhancers and induction of leukemogenesis. Genes Dev 2015; 29: 910–922. 25886910 10.1101/gad.260174.115 PMC4421980

[46] Elliott E , Ezra‐Nevo G , Regev L , Neufeld‐Cohen A , Chen A . Resilience to social stress coincides with functional DNA methylation of the Crf gene in adult mice. Nat Neurosci 2010; 13: 1351–1353. 20890295 10.1038/nn.2642

[47] Zhang X , Odom DT , Koo SH , Conkright MD , Canettieri G , Best J , Chen H , Jenner R , Herbolsheimer E , Jacobsen E , Kadam S , Ecker JR , Emerson B , Hogenesch JB , Unterman T , Young RA , Montminy M . Genome‐wide analysis of cAMP‐response element binding protein occupancy, phosphorylation, and target gene activation in human tissues. Proc Natl Acad Sci USA 2005; 102: 4459–4464. 15753290 10.1073/pnas.0501076102 PMC555478

[48] De Luca LA Jr , Pereira‐Derderian DT , Vendramini RC , David RB , Menani JV . Water deprivation‐induced sodium appetite. Physiol Behav 2010; 100: 535–544. 20226201 10.1016/j.physbeh.2010.02.028

[49] McKinley MJ , Johnson AK . The physiological regulation of thirst and fluid intake. News Physiol Sci 2004; 19: 1–6. 14739394 10.1152/nips.01470.2003

[50] Kao YH , Chen YC , Chung CC , Lien GS , Chen SA , Kuo CC , Chen YJ . Heart failure and angiotensin II modulate atrial Pitx2c promotor methylation. Clin Exp Pharmacol Physiol 2013; 40: 379–384. 23573917 10.1111/1440-1681.12089

[51] Grob M , Trottier JF , Mouginot D . Heterogeneous co‐localization of AT 1A receptor and Fos protein in forebrain neuronal populations responding to acute hydromineral deficit. Brain Res 2004; 996: 81–88. 14670634 10.1016/j.brainres.2003.10.016

[52] Barth SW , Gerstberger R . Differential regulation of angiotensinogen and AT1A receptor mRNA within the rat subfornical organ during dehydration. Brain Res Mol Brain Res 1999; 64: 151–164. 9931478 10.1016/s0169-328x(98)00308-8

[53] Ferguson AV , Renaud LP . Systemic angiotensin acts at subfornical organ to facilitate activity of neurohypophysial neurons. Am J Physiol 1986; 251: R712–R717. 3766770 10.1152/ajpregu.1986.251.4.R712

[54] Thornton SN . Thirst and hydration: physiology and consequences of dysfunction. Physiol Behav 2010; 100: 15–21. 20211637 10.1016/j.physbeh.2010.02.026

[55] Morley JE . Dehydration, hypernatremia, and hyponatremia. Clin Geriatr Med 2015; 31: 389–399. 26195098 10.1016/j.cger.2015.04.007

